# Supplementary material for: Complexome profiling of the Chlamydomonas psb28 mutant reveals TEF5 as an early PSII assembly factor
Source: Plant Cell. 2025 Mar 17;37(6):koaf055. doi: 10.1093/plcell/koaf055 (PMC12164590; doi:10.1093/plcell/koaf055)
Supplement: koaf055_Supplementary_Data [file koaf055_supplementary_data.zip › psb28_interactive.zip!/plots/intensity/qualityPlots/peptides/_Cre13.g572200.t1.2.html]

### Cre13.g572200.t1.2

Trivial name:   
SeqCoverage [median]: 4.30  
Mass: 56.48 kDa  
  
 

| Sequence | MissedCleavage | Mass [Da] | wtIntensity | muIntensity | Score |
| --- | --- | --- | --- | --- | --- |
| AAAGGGVEGAR | 0 | 914.46 | 2014.02 | 813.02 | 97.43 |
| YGSDNVTFTTMAEK | 0 | 1562.69 | 0.00 | 602.11 | 109.72 |
|  |  |  | 2014.0 | 1415.1 |  |
